# Bioactive polysaccharides from natural resources including Chinese medicinal herbs on tissue repair

**DOI:** 10.1186/s13020-018-0166-0

**Published:** 2018-02-06

**Authors:** Qiu Li, Yiming Niu, Panfei Xing, Chunming Wang

**Affiliations:** State Key Laboratory of Quality Research in Chinese Medicine, Institute of Chinese Medical Sciences, University of Macau, Avenida da Universidade, Macau SAR, China

**Keywords:** Polysaccharides, Chinese medicinal herbs, Biomedical applications

## Abstract

**Background:**

Functional polysaccharides can be derived from plants (including herbs), animals and microorganisms. They have been widely used in a broad of biomedical applications, such as immunoregulatory agents or drug delivery vehicles. In the past few years, increasing studies have started to develop natural polysaccharides-based biomaterials for various applications in tissue engineering and regenerative medicine.

**Main body:**

We discuss in this article the emerging applications of natural polysaccharides—particularly those derived from Chinese medicine—for wound healing. First, we introduce natural polysaccharides of three natural sources and their biological activities. Then, we focus on certain natural polysaccharides with growth factor-binding affinities and their inspired polymeric tools, with an emphasis on how these polysaccharides could possibly benefit wound healing. Finally, we report the latest progress in the discovery of polysaccharides from Chinese medicinal herbs with identified activities favouring tissue repair.

**Conclusion:**

Natural polysaccharides with clearly elucidated compositions/structures, identified cellular activities, as well as desirable physical properties have shown the potential to serve as therapeutic tools for tissue regeneration.

## Background

The carbohydrates, along with proteins, lipids and nucleic acids, are regarded as the major biomacromolecules. Most carbohydrates found in nature existing as polysaccharides are composed of monosaccharides [1], which can be found in almost all kingdoms of life, such as the algae [2] (e.g. alginate), plants [3] (e.g. starch and cellulose), microbes [4] (e.g. zymosan and dextran) and animals [5] (e.g. hyaluronic acid and heparin). Polysaccharides can be classified according to their electric charge: cationic polysaccharide [6] (chitin, chitosan), anionic polysaccharide [7, 8] (heparin, hyaluronic acid, alginic acid and chondroitin sulfate) and nonionic polysaccharide [9] (dextran, starch, and cellulose). In terms of chemical composition, polysaccharides can also be classified as the homo-polysaccharide [10] containing a single type of monosaccharide and hetero-polysaccharide [11] containing two or more different types of monosaccharides. For example, cellulose consists of unique glucose and heparin consists of the α-l-idopyranosyluronic acid 2-sulfate and 2-deoxy-2-sulfoamino-α-d-glucopyranose 6-sulfate [12]. Based on the different glycosides linked with glycan, polysaccharides also exist in the forms of proteoglycans, glycolipids and glycoconjugates. The rapid development of bioanalytical technology has enabled to understand the structure of polysaccharides and utilise their functions. Polysaccharides, together with oligosaccharides, not only serve as the building blocks of the life but also mediate many biological signals, including cell–cell communication [13], immune recognition [14], and mitogenesis [15].

These findings motivate the development of polysaccharides for biomedical applications—as therapeutic agents, drug carriers and tissue scaffolds. For instance, starch and glycogen have long been used as biofuels [16], adjuvants and food additives [17]. More applications of polysaccharides are also inspired by their native functions in constructing the extracellular matrix and supporting cell adhesion and proliferation [18]. However, unlike proteins that can be more accurately characterised and ‘bottom-up’ synthesised, polysaccharides are generally very difficult to characterise for a precise structure, based on the current technical conditions. Polysaccharides have diverse chemical structure, composition, molecular weight, potential and linking sequence, all of which result in different functionality and biological activity [19]. Therefore, it is both interesting and challenging to obtain novel, functional polysaccharides and elucidate the relationship between its structure and activity. Successful understanding of the mechanism of biological effects requires multidisciplinary knowledge and various technologies. Here, we concisely review the previous research into naturally derived functional polysaccharides in biomedical science, and discuss the potential of those derived from Chinese medicine in tissue regeneration, which may represent a promising direction in this field of research [20–28] (Table 1).

**Table 1:** Various polysaccharides in nature

Polysaccharides	Source	Functions
Starch	Plants	Storage, drug adjuvant
Cellulose	Plants	Cell structure, food additives
Pectin	Plants	Food additives
Alginate	Microorganism	Drug adjuvant
Carrageenan	Microorganism	Food additives
Heparin	Animals	Animal tissue structure, therapeutic agents
Hyaluronan	Animals	Animal tissue structure, therapeutic agents
Chondroitin sulfate	Animals	Animal tissue structure
Heparin sulfate	Animals	Animal tissue structure
Chitin and chitosan	Animals	Tissue scaffolds

### Plant polysaccharides: biological activities and biomedical applications

In the past decades, the polysaccharides derived from herbs, such as various Chinese medicines, have attracted much attention in a multiple of fields. Numerous researches indicate that polysaccharides can be used in many fields and have a diverse of therapeutic properties, such as antioxidant activity [29], antitumor activity [30], the effect of promoting wound healing [31] and immunostimulatory activity [32].

Firstly, plant polysaccharides have been used for industrial applications, e.g. pharmaceuticals, biomaterials, food stuff and nutrition, and biofuels. For example, a variety of indigestible plant polysaccharides including cellulose, hemicelluloses, pectins, oligosaccharides, gums, was defined as the dietary fiber by the Food and Agriculture Organization (FAO). Among these, cellulose and hemicellulose can directly stimulate the bowel movement, which is the most widely spreading polymeric material in nature, is a fibrous, tough, water-insoluble material. The cellulose commonly found in the cell walls of plants-stalks, stems or trunks, is a linear polysaccharide consisting of β-d-glucan units linked by (1 → 4) glycosidic bonds [33]. The materials based on the cellulose have been extensively used in biomedical field [34], such as the adsorbent beads, filter, artificial tissue, and protective clothing. Among these applications, the cellulose due to the mechanical strength and biocompatibility, can be applied for tissue engineering [35], including engineering vascular tissue, and a series of other tissues, such as bone, cartilage, skeletal muscle, cardiac muscle, and heart valves. Additionally, cellulose has also been used to establish nano-fibrous carrier for liver cells and create tubes for regeneration of damaged peripheral nerves. Gu performed the research of creating carriers for delivery and differentiation of mesenchymal stem cells [36]. However, the applications of the cellulose are limited by the solubility in common organic solvents. It is difficult to melt due to the strong intermolecular and intramolecular hydrogen bonds.

Secondly, herbs have been used to treat kinds of illnesses and modern pharmacological experiments have identified that the main or key components of herbal medicines generally include much ingredients. Of these fractions in herbal medicines, polysaccharides have been identified as major active ingredients, responsible for various pharmacological activities. Although the detailed mechanism of these effects is under exploration, the immunostimulatory activities of many polysaccharides are confirmed. It appears that immune cells, especially macrophages [3], involve this regulating process. Macrophages play a vital role in kinds of complex microbicidal functions, including the surveillance [37], chemotaxis [38], phagocytosis [39], and degradation of the target organisms. And polysaccharides can modulate the function of macrophages. The studies about the effects of plant polysaccharides on macrophage functions have demonstrated that glycan can enhance macrophage functions, which include activating phagocytic ability [40], increasing the cytotoxic activity against the tumour cells, reactive oxygen species (ROS) and nitric oxide (NO) production, and secretion of cytokines and chemokines, such as tumour necrosis factor (TNF-α), interleukin-1β (IL-1β), IL-6, IL-12 and so on [41]. For example, Schepetkin and Quinn extracted a polysaccharide from the cones of *Juniperus scopulorum*, which composed of arabinogalactan, and showed significant immunomodulatory effect to the murine macrophages [42]. It was certified that the polysaccharide caused the increasing expression of macrophage iNOS and NO, enhanced secretion of cytokines like IL-1, IL-6, IL-12, IL-10 and TNF-α. Popov and Ovodov isolated and obtained a pectic polysaccharide from *Silene vulgaris*, which can enhance the myeloperoxidase activity of macrophage via extracellular Ca^2+^, whereas, the polysaccharide obtained from the same species can show the same effect without the extracellular Ca^2+^. We can conclude that the polysaccharides from the same plant may induce different signal transductions. Luk found that polysaccharides extracted from the *Tripterygium wilfordii* exhibited the effect of suppressing the secretion of TNF-α and expression of some proteins (CD11c, CD18, CD14 and CD54) in human monocytic THP-1 cells [43, 44]. Additionally, polysaccharides derived from herbs can also induce macrophage hematopoiesis [3]. Song found that polysaccharides from *Chelidonium majus* could increase the amount of granulocyte–macrophage colony-forming cells in experimental animals [45]. Meanwhile, a polysaccharide from *Aloe barbadensis* also showed significant hematopoietic effect and induced production of monocytes [46].

We found that polysaccharides from plants activate macrophages mainly via the interaction with specific receptors on cells, which are usually known as pattern recognition receptors. Macrophages could bind and interact with the polysaccharides through toll-like receptor 4 (TLR4) [47], CD14 [48], dectin-1 [49] and mannose receptor [50], among others. After the activation of the receptors, it can lead to downstream signal and production of pro-inflammatory factors. Ando and Kataoka found that polysaccharides extracted from *Carthamus tinctorius* could active the macrophage through TLR4, inducing downstream signals and expression of TNF-α and NO [51]. Further, the test in vitro was performed in peritoneal macrophage from the C3H/HeJ mice which have a point mutation in the TLR4 gene and the activating effect disappeared. Moreover, regarding the polysaccharides from the roots of *Astragalus membranaceus*, the results also showed that the relative response disappeared in the peritoneal macrophage from the C3H/HeJ mice [52]. It suggested that the TLR4 receptor involved the signal transaction of macrophage activation.

**Fig. 1:**
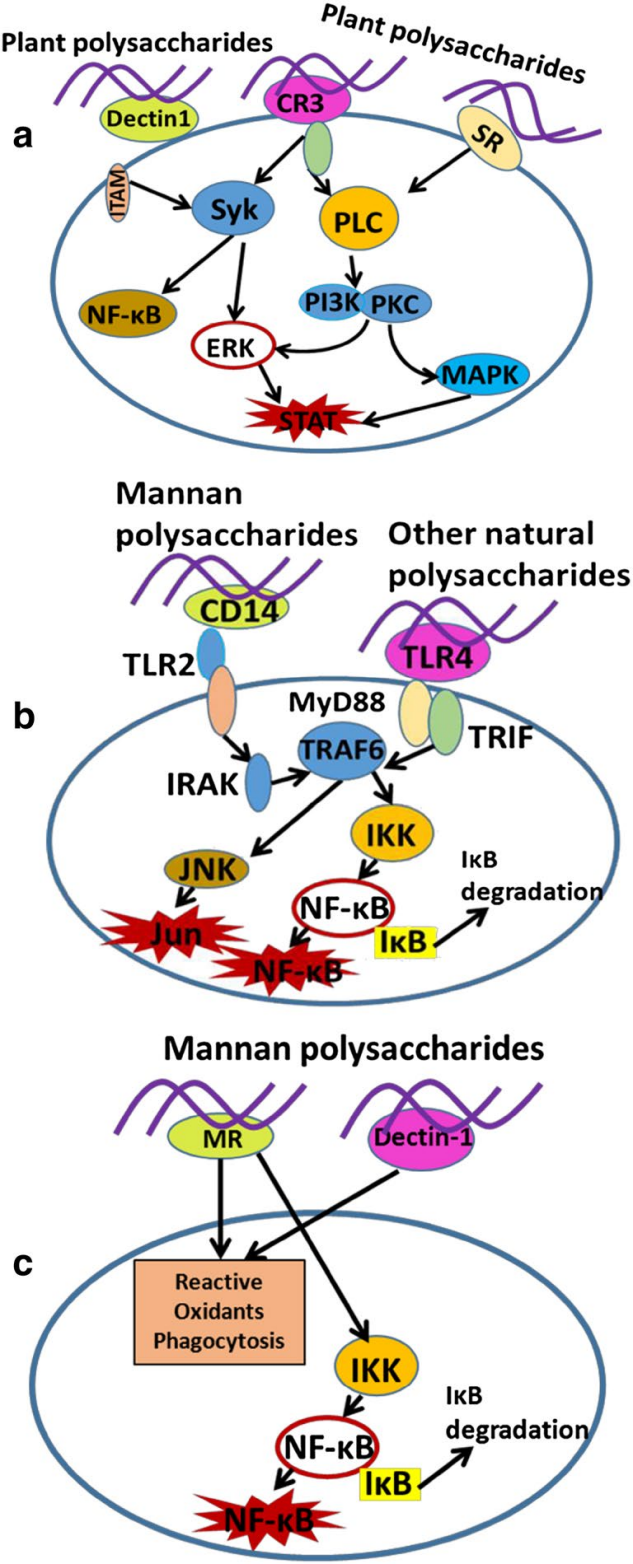
**a**–**c** Potential signalling pathways involved in macrophage activation by plant polysaccharides [3, 53]

In summary, carbohydrates may interact with macrophages and regulate these cells in diverse mechanisms, some of which are shown in Fig. 1. For example, carbohydrates interact with the TLR4/MD-2 complex resulting in dimerisation of two TLR4/MD-2 complexes, recruitment of two adaptor molecules, MyD88 and TRIF, and activation of intracellular signalling pathways (NF-κB) [53]. The mannose receptor is also a potential receptor inducing macrophage phagocytosis, endocytosis and NF-κB signalling pathways. Additionally, CR3 is involved in the activation of phosphoinositide-3-kinase (PI3K), the mitogen-activated protein kinase (MAPK) and NF-κB signalling pathways (Table 2; [54–60]).

**Table 2:** The polysaccharides derived from herbs in nature

Polysaccharides	Composition	Source	Physiological effects
Cellulose	β-(1-4)-Linked-glucopyranose	Grains, fruit, vegetables	Cell structure, food additives, regulate bowel movement
*Juniperus scopolorum* polysaccharide	β-Galactopyranose, and α-arabinofuranose	*Juniperus scopulorum*	Immunomodulatory effect to the murine macrophages
*Konjac glucomannan* polysaccharide	β-(1-4)-Linked-glucose, β-(1-4)-linked-mannose	*Amorphophallus konjac* plant	Cholesterol lowering and immunoregulation
*Chelidonium majus* polysaccharide	Galactose, mannose, glucose in the molar ratio of 5:4:1	*Chelidonium majus*	An effective antitumor immunostimulator
*Reishi* polysaccharide	Arabinose, rhamnose, xylose, mannose, glucose at the different ratios	*Ganoderma lucidum*	Stimulating the expression of inflammatory cytokines
*Ginseng* polysaccharide	(1-4)-Linked homogalacturonan backbone	*Ginseng*, the root of *Panax ginseng*	Anti-rotavirus activity
*Bletilla striata* polysaccharide	α-Mannose, β-mannose and β-glucose at the ratio of 2.4:1	*Bletilla striata*	Modulating the function of macrophages
*Eucommia ulmoides* polysaccharide	Mannose, galactose, glucose, arabinose, rhamnose, and galacturonic acid	*Eucommia ulmoides*	Binding PDGF-BB growth factor and anti-inflammatory effect
*Astragalus* polysaccharides	Rhamnose, arabinose and glucose in a molar ratio of 1:6.25:17.86	The roots of *Astragalus*	The effect of immunomodulatory
Pectin	α-(1-4)-d-Galacturonic acid and rhamnose	Plant primary cell wall	Food additives

### Animal-derived polysaccharides: potential biomedical functions

Except for functional polysaccharides from herbs, the polysaccharides derived from animals also play a vital role as tissue composition and exhibit significant effect in biomedical science. Among these, extracellular matrix in animal tissues, composed of an interlocking meshwork of heteropolysaccharides and fibrous proteins, is filled with a gel-like material, which supports cell adhesion, growth and provides a porous pathway for the diffusion of nutrients and oxygen to individual cells [61]. For instance, the heteropolysaccharides, called glycosaminoglycans, are a family of linear polymers composed of repeating disaccharide units [62]. Glycosaminoglycans (GAGs) include hyaluronic acid, heparin and heparan sulfate, chondroitin sulfate (CS), dermatan sulfate, keratin sulfate. In addition to GAGs, the chitosan and chitin also belong to animal derived polysaccharides, which are widely used in biomedical science.

#### Heparin and heparan sulfate

Heparin possessing highly sulfated, linear structure is an important member of glycosaminoglycans (GAGs), which consists in repeated units of sulfonated hexuronic acid (1 → 4) d-glucosamine. The residue of uronic acid in heparin consisting of α-l-iduronic acid (IdoA) or β-d-glucuronic acid (GlcA) can be sulfated at 2-*O* position. The residue of glucosamine can present unmodified (GlcN), *N*-sulfonated (GlcNS), or *N*-acetylated (GlcNAc), with various *O*-sulfations at the 3-*O* and 6-*O* positions [63]. And heparan sulfate has a higher ratio of IdoA residues and sulfate groups.

Heparin can be biosynthesized and stored in mast cells, whereas heparan sulfate as a proteoglycan, mainly exists on the surface of cells and in extracellular matrix of tissue. Heparin widely used as one of the oldest drug in clinic plays part in many physiological and pathophysiological processes, such as angiogenesis, cell adhesion, cell growth, inflammation and anticoagulation [64, 65]. Numerous researches indicated that heparin could modulate the relative biological processes via binding with the basic amino acid groups of proteins, such as binding with growth factors [66], forming the complex to stabilize growth factors and prolong the function of growth factors. For example, Shah and Revzin prepared bioactive heparin-based hydrogel systems, which showed sustained release of hepatocyte growth factor [67]. The common function of heparin is the anticoagulation activity which induce the effect by interacting with the serine protease inhibitor antithrombin III [68]. With regard to heparan sulfate, it is reported that heparan sulfate on the cell surface serves as the receptors of adhesion for many bacterial pathogens [69].

Although heparin as clinical drug has been used for treating venous thrombosis, pulmonary embolism, and acute coronary syndrome [70], there are still some limitations including having the risks of potential bleeding and heparin-induced thrombocytopenia [71]. Thus, the low molecular weight heparin obtained from the unfractionated heparin were developed into therapeutic agent to alleviating side effects in clinic [72]. Certainly, more understanding of the structure–activity relationship is required in the relative biological processes. We can further explore the drugs derived from heparin and heparan sulfate to improve therapeutic effect in clinic.

#### Hyaluronic acid

The hyaluronic acid owning high molecular weight [73], which is an anionic and non-sulfate polysaccharide and consists of alternating units of d-glucuronic acid, and *N*-acetyl-d-glucosamine, is the component of ECM. It was discovered by Karl Meyer and his assistant, John Palmer in 1934. The hyaluronic acid is a naturally substance which is naturally existing in our body, and has crucial biological functions in our body [74, 75]. In human body, it is found in most connective tissues, especially in the eyes and joints. The use of hyaluronic acid in some eye surgeries including the cataract removal, corneal transplantation, and repair of a detached retina and other eye injuries, has been approved by FDA. It can be injected into the eye during the process to aid instead natural fluids [76].

According to numerous studies, hyaluronic acid (HA) owns various biological activities. It has chondroprotective effects in vivo and can evidently influence on the articular cartilage [77]. It was reported that exogenous HA could promote the synthesis of proteoglycan, modulate the functions of immune cells, and reduce the activity of proinflammatory cytokines [78]. Additionally, HA has the prominent ability of water retention and play a vital role in regulating tissue hydration and osmotic balance [79]. Because of the highly hygroscopicity, HA can significantly regulate the physical properties of ECM. Hyaluronic acid with special structure is usually considered as a prominent signalling molecule, which can interact with cell surface receptors and thereby modulate cell adhesion, migration, and proliferation [80, 81]. Among these signals, CD44 can bind with HA and the relative signal pathways were confirmed in hematopoietic cells from CD44-deficient mice [82]. The results suggested that there are CD44-independent mechanisms for the guidance of gene expression by HA. Above all, HA has been widely used for more than 20 years over the world due to the biocompatibility and biodegradability. Scientists have made great progress of HA applications in biomedical science. However, the mechanism of HA interacting with the cells need to be explored more clearly and the clinical application of the material derived from HA still has a long way to go.

#### Chitosan and chitin

Chitosan, the deacetylated derivative of chitin obtained from arthropods usually exists in the form of granules, sheets, or powders. Chitosan and chitin are both linear polysaccharides, composed of the repeated units of N-acetyl-2-amino-2-deoxy-D-glucose (N-acetylated groups) and 2-amino-2-deoxy-D-glucose residues (N-deacetylated groups, amino groups). Chitosan as a heteropolysaccharide also includes linear β-1,4-linked units [83]. Numerous studies suggest that chitosan and chitin can be used for various applications in tissue engineering [84], like wound healing, and drug delivery [85]. They can be engineered versatile formations such as the gels [86], membranes [87], nanofibers, nanoparticles [88], and sponges [89]. At present, many studies trying to develop the chitinous scaffolds in tissue engineering were reported and have made great progress [90]. These chitosan scaffolds exhibited the significant effect on supporting and aiding the generation of extracellular matrix containing abundant proteoglycan in vivo. Additionally, the chitosan and chitin are also widely used as the skin substitutes in tissue engineering [91]. It attributes to the excellent properties of chitin including hemostasis and biocompatible [92], which can facilitate tissue regeneration and generation of the extracellular matrix. And the chitosan was also demonstrated that it could promote wound healing via accelerating the infiltration of polymorphonuclear (PMN) cells at the wound site [93].

### Polysaccharides derived from microorganism: functions and applications

Polysaccharides derived from the microorganism are also one class of the major polysaccharides existed in nature. Microbial polysaccharides may be neutral (e.g. dextran, scleroglucan) or acidic (xanthan, gellan) in nature. Some of these polysaccharides such as the glycogen serve as storage compound. Moreover, microbial polysaccharides present a great potential for medical, pharmaceutical and biomedical applications, such as wound dressings, biomaterials, and tissue regeneration.

#### Alginate

Alginate is a class of naturally existing anionic polymer, which can be extracted from brown algae cell walls, including *Macrocystis pyrifera*, *Laminaria hyperborea*, *Ascophyllum nodosum*. Alginate is a linear polysaccharide composing of the repeated units of 1,4-linked β-d-mannuronate (M) and 1,4-α-l-guluronate residues (G). The common alginate usually derived from algal owns highly different physical–chemical heterogeneity which can affect their quality and induce different applications [94]. The alginate possessing kinds of outstanding properties has been extensively studied for biomedical applications [95], including their biocompatibility, low toxicity, low cost, and moderate gelation induced by divalent cations such as Ca^2+^.

The alginate gel formed by the inducing of divalent cations can be used for wound healing [96], therapeutic agents, proteins delivery [97], and cell transplantation [98]. The wound dressing made by alginate can stimulate the extracellular matrix and establish a moist environment, which could decrease the risk of bacterial infection at the injured site, and accelerate the wound healing rate. Drug and protein systems which can deliver bioactive agents and biomacromolecules, were fabricated by alginate, and could release bioactive molecules in a controlled manner. The alginate gels are also applied for cell transplantation in tissue engineering [99]. It can deliver cells to the designated site, providing artificial matrix for neovascularization. Additionally, the alginate gels can also be orally administrated or injected into body, which can be used in pharmaceutical filed [100].

#### Dextran

Dextran, a high molecular-weight polysaccharide, composed of α-1,6 linking glucose of the backbone, α-1,4 linking glucose of side chain. The dextran extracted from different microbial strain possesses different structures [101]. After the crosslinking of the dextran, it usually can be used for the separation and purification of biomacromolecules. Due to its biocompatibility, it also can be applied as the plasma expander for biomedical application (Table 3; [102, 103]).

**Table 3:** The different kinds of non-plant-origin polysaccharides in nature

Polysaccharides	Composition	Source	Physiological effects
Alginate	Repeated units of 1,4-linked β-mannuronate and 1,4-α-guluronate residues	Brown algae (Phaeophyceae)	Wound healing, therapeutic agents and proteins delivery, and cell transplantation
Carrageenan	Repeating galactose units and 3,6 anhydrogalactose	Red edible seaweeds	Food additives and immunoregulatory effect
Mushroom polysaccharides	β-Glucans and heteropolysaccharides	Mushrooms	Antiobesity, antidiabetes, anticancer, and antibiotic properties
Heparin	Repeated units of sulfonated hexuronic acid (1 → 4)-glucosamine	Porcine intestinal mucosa	Animal tissue structure, binding affinity for growth factors, and anticoagulation
Hyaluronan	Alternating units of d-glucuronic acid, and *N*-acetyl-d-glucosamine	Synovial fluid, the vitreous fluid of the eye, umbilical cords and chicken combs	Natural fluids, wound dressing, chondroprotective effects
Chondroitin sulfate	Alternating sugars (*N*-acetylgalactosamine and glucuronic acid)	Cartilage of animals	Animal tissue structure, dietary supplement for treatment of osteoarthritis
Heparin sulfate	A glucuronic acid (GlcA) linked to *N*-acetylglucosamine	Animals	Animal tissue structure
Chitin and chitosan	Repeated units of *N*-acetyl-2-amino-2-deoxyd-glucose and 2-amino-2-deoxy-dglucose residues	Crab or shrimp shells and fungal mycelia	Wound healing, and drug delivery

### Polysaccharides to enrich growth factors for wound healing

Traumatic injury is a leading cause of mortality in many countries. Accelerating the healing, while minimising the aesthetic impact on patients and restoring full functions the tissue, remains an unmet clinical goal. Although minor injuries in healthy bodies generally heal, the healing of large injuries is often hampered by many factors, such as the age of the patient, infection at the wound site and chronic diseases. The detailed mechanisms are poorly understood.

To most organs and tissues, wound healing has three overlapping stages: inflammation, proliferation and remodelling. In all stages—in particular the second and third, multiple families of growth factors play essential, diverse and co-ordinated roles. For example, several members in the vascular endothelial growth factor (VEGF) and fibroblast growth factor (FGF) families are primary mediators of angiogenesis, while several FGFs also direct fibroblast proliferation and migrations. The platelet-derived growth factor family (PDGF), in particular the PDGF-BB variant, is required for vascular maturation; while transforming growth factors (TGFs) regulate the collagen synthesis. Importantly, many growth factors are bound and protected by glycosaminoglycans (GAGs)—which are anionic, sulphated polysaccharides—in mammalian tissues. In the absence of GAGs, the growth factors cannot be enriched and may easily be degraded or diffused.

This feature provides an exciting opportunity for the design of growth factor-binding polysaccharides for wound healing, which, compared with conventional polysaccharides scaffolds as dressing, possess clearer and more specific bioactivities. Numerous attempts have been performed for engineering polysaccharide scaffolds to bind and enrich growth factors, which showed better effect on wound repair than free growth factors. For example, researchers fabricated a heparin-based hydrogel consisting of thiolated heparin and diacrylated poly (ethylene glycol) using photo polymerisation, which was loaded with human epidermal growth factor (hEGF) for skin repair in mice. It showed a sustained release profile of hEGF in vitro and an accelerated healing of skin incision in vivo, in comparison with using free hEGF alone [104]. In another study, Wu and Xiao explored a heparin-based coacervate composed of poly (ethylene argininylaspartate digylceride) (PEAD) as a reservoir, heparin as a bridge, and fibroblast growth factor-2 (FGF-2) as a cargo. The regenerative effect of this scaffold was evaluated in mice with full-thickness excisional skin wounds. The results indicated that this coacervate exhibited faster wound closure, compared with the control and free FGF-2 groups [105]. Additionally, numerous synthetic polymers are devised to mimic the action of GAGs. For instance, a supramolecular polyelectrolyte complexation with sulfonated polyrotaxanes (PRXs) loaded with bone morphogenetic protein 2 (BMP-2) was fabricated, which enhanced the osteogenetic differentiation of BMP-2 in vitro and improved the healing of a calvarial defect in mice [106]. A more direct approach, as demonstrated by Wang and colleagues, was to isolate a fraction from heparan sulfate, named HS^7+^, which had a higher binding affinity for VEGF-A than the crude sugars. The results in vitro and in vivo demonstrated the potential for vascular therapy of HS^7+^ targeted at enhancing the bioactivity of VEGF-A [107].

### Polysaccharides from Chinese medicinal herbs—new prospects in tissue engineering

In the past decades, lots of polysaccharides were obtained from Chinese medicinal herbs; there are many polysaccharides that have received massive attention as promising biomaterials for various applications because of their biocompatible, safe and biodegradable properties. As summarized by above, polysaccharides from Chinese medicinal herbs have comprehensive physiological activity in human body. Meanwhile, much studied indicated that polysaccharides play a vital role in regulating the immune system. However, few attentions focus on the development of polysaccharides from Chinese medicinal herbs as functional biomaterials in tissue engineering.

As mentioned above, macrophages can be activated by many polysaccharides in the nature through relatively specific membrane receptors. The interactions between certain saccharide units (e.g. mannose, β-glucan) and receptors induce multiple cellular responses. According to this property, the polysaccharides can be developed into biomaterial matrices for tissue engineering. For example, as a traditional Chinese medicine, *Bletilla striata* has long been applied for stop bleeding and wound healing. The *B. striata* polysaccharide is obtained from *B. striata*, which composed of α-mannose, β-mannose and β-glucose at the mole ratio of 2.4:1. It can be widely used in tissue regeneration. Luo found that after the wound treated with BSP gel, it was proved to control the inflammatory responses and accelerate the wound closure [108]. In another study, the cotton gauze coated with aqueous extract of *B. striata* polysaccharide showed better healing effect [109].

Further, *B. striata* polysaccharide (BSP), as a typical glucomannan, derived from a Chinese medicinal herb was studied for its bioactivity in modulating macrophages. It was demonstrated that BSP could modulate the function of macrophages via binding mannose receptor and regulating downstream signals [110]. This polysaccharide also could active macrophages and regulates the secretion of cytokines for regeneration of engineered tissues. Accordingly, Niu found a polysaccharide from *B. striata*, which can be used for modulating the phenotype of host macrophage after the acetylation of this polysaccharide. The results suggested that the polysaccharide can stimulate macrophages into a pro-osteogenic phenotype; further, the scaffold manufactured by the polysaccharide had a competent ability as an innovative and efficacious platform to harness the power of host immunity for enhancing the regenerative performance of engineered tissue constructs. This research innovatively modulates the function of macrophages in tissue engineering.

Except for BSP, another polysaccharide, derived from *Konjac*, also is a glucomannan, which can be used for wound healing due to its excellent physical and chemical properties. Fan fabricated a film of a blend of *Konjac* glucomannan (KGM) and chitosan. It was indicated that this film showed a strong antibiotic effect and haemostatic efficiency compared with conventional materials [111]. Meanwhile, Feng found that the natural polysaccharide form *Konjac* glucomannan had affinitive for macrophages, and stimulate them to release growth factors and cytokines [112]. They further modified this polysaccharide with heparin, and then designed an injectable hydrogel scaffold composed of KGM polysaccharide and heparin. They evaluated the efficacy of this scaffold in promoting angiogenesis in situ. The results demonstrated that this scaffold based on polysaccharides had a prominent potential in regenerative medicine.

It is concluded that the glucomannan can interact with mannose receptor. As mentioned in the last section, it is an interesting direction to discover carbohydrates of non-animal source with growth factor-binding ability. Accordingly, Li obtained a polysaccharide from *Eucommia ulmoides*, named EUP3, containing a proportion of galacturonic acid [58]. Unlike animal derived polysaccharides—glycosaminoglycans binding various growth factors, EUP3 polysaccharide had no significant affinity for VEGF-A and FGF-2, but had a clear affinity for PDGF-BB. Further, Li developed EUP3 into a growth factor-affinitive scaffold using electrospinning technology [113]. The results indicated that this scaffold based on EUP3 polysaccharide could accelerate angiogenesis and promote wound healing via sequestering PDGF-BB growth factor.

As we concluded above, polysaccharides from the Chinese medicinal herbs have a promising potential for the applications in tissue engineering. Numerous researches have been performed for engineering suitable polysaccharide scaffolds via interdisciplinary biotechnologies. Above all, compared with polysaccharides from animals, which are often amorphous and have weakly mechanical properties, the polysaccharides from Chinese medicinal herbs have better mechanical properties. Moreover, the polysaccharides from animals often have the risk of immune response and other side effects. Therefore, because of the limitations of animal-derived polysaccharides, it has a promising prospect that screening the polysaccharides from the Chinese medicinal herbs, which have specific properties, can be applied in tissue engineering.

## Conclusions

Polysaccharides are natural biomaterials which are inexpensive, and most of them are easily obtained. The special structure diversities and physiochemical properties of polysaccharides can be exerted successfully, and lots of polysaccharides have been developed into functional biomaterial matrices. In sum, they have been applied in tissue engineering by mainly three approaches: (1) compatible materials for tissue regeneration, (2) drug delivery materials, and (3) immunoregulatory agents.

However, although lots of polysaccharides were obtained and various bioactivities of polysaccharides were applied in tissue engineering, the mechanism of polysaccharides interacting with bodies was still unclear due to the complicated structures. The detailed mechanisms and structure–activity relationship should be studied further. Moreover, it is a challenge that obtains the functional polysaccharides with high purity and characterizes the structure of polysaccharides. Additionally, in terms of the application of functional polysaccharides in tissue regeneration, although numerous researches have been carried out for developing the functional scaffolds, there is still a long way to transform from research to clinic. At present, there are still many limitations which include the immunogenicity of scaffolds, high cost and high failure rate. Developing the ideal polysaccharide scaffolds, which can be used in clinic, must satisfy these requirements: the clear structure of polysaccharides; definite bioactivities, security and biocompatibility, and appropriately physico-chemical properties. We suppose that the Chinese medicinal herbs could be a potential and abundant natural source for developing promising biomaterials in future.

## Abbreviations

ROS: reactive oxygen species
NO: nitric oxide
TNF-α: tumour necrosis factor
IL-1β: interleukin-1β
HA: hyaluronic acid
GAGs: glycosaminoglycans
ECM: extracellular matrix
